# Validation of impaired Transient Receptor Potential Melastatin 3 ion channel activity in natural killer cells from Chronic Fatigue Syndrome/ Myalgic Encephalomyelitis patients

**DOI:** 10.1186/s10020-019-0083-4

**Published:** 2019-04-23

**Authors:** H. Cabanas, K. Muraki, C. Balinas, N. Eaton-Fitch, D. Staines, S. Marshall-Gradisnik

**Affiliations:** 1 School of Medical Science, Griffith University, Gold Coast, QLD Australia; 2 The National Centre for Neuroimmunology and Emerging Diseases, Menzies Health Institute Queensland, Griffith University, Gold Coast, Southport, QLD 4222 Australia; 3 Laboratory of Cellular Pharmacology, School of Pharmacy, Aichi-Gakuin University, Chikusa, Nagoya, Japan; 4 Consortium Health International for Myalgic Encephalomyelitis, National Centre for Neuroimmunology and Emerging Diseases, Griffith University, Gold Coast, QLD Australia

**Keywords:** Transient receptor potential Melastatin 3, Calcium, Chronic fatigue syndrome/Myalgic encephalomyelitis, Natural killer cells, Patch-clamp

## Abstract

**Background:**

Chronic Fatigue Syndrome/ Myalgic Encephalomyelitis (CFS/ME) is a complex multifactorial disorder of unknown cause having multi-system manifestations. Although the aetiology of CFS/ME remains elusive, immunological dysfunction and more particularly reduced cytotoxic activity in natural killer (NK) cells is the most consistent laboratory finding. The Transient Receptor Potential (TRP) superfamily of cation channels play a pivotal role in the pathophysiology of immune diseases and are therefore potential therapeutic targets. We have previously identified single nucleotide polymorphisms in *TRP* genes in peripheral NK cells from CFS/ME patients. We have also described biochemical pathway changes and calcium signaling perturbations in NK cells from CFS/ME patients. Notably, we have previously reported a decrease of TRP cation channel subfamily melastatin member 3 (TRPM3) function in NK cells isolated from CFS/ME patients compared with healthy controls after modulation with pregnenolone sulfate and ononetin using a patch-clamp technique. In the present study, we aim to confirm the previous results describing an impaired TRPM3 activity in a new cohort of CFS/ME patients using a whole cell patch-clamp technique after modulation with reversible TRPM3 agonists, pregnenolone sulfate and nifedipine, and an effective TRPM3 antagonist, ononetin. Indeed, no formal research has commented on using pregnenolone sulfate or nifedipine to treat CFS/ME patients while there is evidence that clinicians prescribe calcium channel blockers to improve different symptoms.

**Methods:**

Whole-cell patch-clamp technique was used to measure TRPM3 activity in isolated NK cells from twelve age- and sex-matched healthy controls and CFS/ME patients, after activation with pregnenolone sulfate and nifedipine and inhibition with ononetin.

**Results:**

We confirmed a significant reduction in amplitude of TRPM3 currents after pregnenolone sulfate stimulation in isolated NK cells from another cohort of CFS/ME patients compared with healthy controls. The pregnenolone sulfate-evoked ionic currents through TRPM3 channels were again significantly modulated by ononetin in isolated NK cells from healthy controls compared with CFS/ME patients. In addition, we used nifedipine, another reversible TRPM3 agonist to support the previous findings and found similar results confirming a significant loss of the TRPM3 channel activity in CFS/ME patients.

**Conclusions:**

Impaired TRPM3 activity was validated in NK cells isolated from CFS/ME patients using different pharmacological tools and whole-cell patch-clamp technique as the gold standard for ion channel research. This investigation further helps to establish TRPM3 channels as a prognostic marker and/ or a potential therapeutic target for CFS/ME.

## Background

Chronic Fatigue Syndrome/ Myalgic Encephalomyelitis (CFS/ME) is a complex multi-faceted illness characterized by persistent, debilitating fatigue that is unrelieved by rest (Fukuda et al., 1994). This is accompanied by a variety of other adverse symptoms including: cognitive, muscular, joint, gastrointestinal, neurological and cardiovascular impairments (Carruthers et al., 2011). The severity of these symptoms varies between patients; ranging from mild to severe. However, there are currently neither standardized diagnostic laboratory tools nor targeted treatments available. Diagnosis instead relies on the use of multiple symptom-specific case definitions that describes features of the illness following exclusion of all other potential clinical causes (Carruthers et al., 2011; Jason et al., 2015). The Fukuda case definition (or Centers for Disease Control and Prevention (CDC) criteria (1994)) was the first set of frameworks established that are widely used to define CFS/ME populations both in research and clinical practice (Fukuda et al., 1994). However, this definition has been considered too broad in its symptoms requirements for a definite diagnosis (Johnston et al., 2013). More specific frameworks such as the Canadian Consensus Criteria (CCC) (2003) and International Case Criteria (ICC) (2011) have been developed to improve case identification (Carruthers et al., 2011).

While the aetiology underlying CFS/ME symptom manifestation is not well understood, immunological disruption, in particular, a significant reduction in Natural Killer (NK) cell quantity and cytotoxic activity is consistently described (Brenu et al., 2011a; Brenu et al., 2012; Curriu et al., 2013; Hardcastle et al., 2015; Huth et al., 2016; Klimas et al., 1990; Maher et al., 2005; Natelson et al., 2002; Nijs & Frémont, 2008; Sharpe et al., 1991; Siegel et al., 2006; Stanietsky & Mandelboim, 2010). NK cells are innate lymphoid cells that are involved in the destruction of tumor and virally- infected cells. This cell type also has important roles in the production of cytokines and immune cell activation (Vivier et al., 2008). NK cell phenotype is determined through the expression of cell-surface markers including CD56 and CD16. The most commonly found combination is: CD56^dim^CD16^bright^ which constitutes 90% of the human peripheral NK cells and exhibits high cytotoxic activity (Cooper et al., 2001). Multiple NK cellular processes including: adhesion to target cell, release of lytic proteins, formation of perforin pores and granzyme-induced apoptosis are dependent on tight regulation of calcium (Ca^2+^) signalling (Anasetti et al., 1987; Henkart, 1985; Kass & Orrenius, 1999; Schwarz et al., 1833).

Regulation of Ca^2+^ processes is complex and is orchestrated by a variety of different cellular and soluble components (Berridge, 2012). One mediator of particular interest is the large and diverse family of Transient Receptor Potential (TRP) non selective cation channels, which function as polymodal cellular sensors involved in the fine-tuning of many biological processes in both excitable and nonexcitable cells (Gees et al., 2010). Intriguingly, mutations in the encoding genes are associated with a plethora of diseases, and TRP channels are considered potential channel therapeutic targets (Nilius et al., 2007). On the basis of sequence homology and modular domain structure, the mammalian TRP family can be further divided into six subfamilies: TRPC (canonical), TRPM (melastatin), TRPV (vanilloid), TRPA (ankyrin), TRPML (mucolipin), and TRPP (polycystin) (Clapham et al., 2001). The TRPM subfamily includes the Transient receptor potential melastatin 3 (*TRPM3) channel*, which is a Ca^2+^-*permeable* nonselective cation channel widely expressed in many different tissues and cell types including adipocytes, pancreatic beta-cells, the kidney, eye, brain and the pituitary gland (Vriens et al., 2011; Hoffmann et al., 2010; Oberwinkler & Philipp, 2014; Wagner et al., 2008; Thiel et al., 2013). TRPM3 has been found to serve many different functions including secretion of factors (e.g. insulin and interleukin-6), vascular contraction, heat-sensing, and zinc influx (Vriens et al., 2011; Wagner et al., 2008; Naylor et al., 2010). TRPM3 is a typical TRP cationic channel containing six transmembrane domains and a pore domain between the fifth and sixth transmembrane domain. Both amino and carboxy termini are located in the cytosol (Venkatachalam & Montell, 2007). Whereas several splice isoforms have been identified, the TRPM3α2 isoform (TRPM3–9 in human) is by far the best characterized and known as highly permeable for Ca^2+^ (Oberwinkler et al., 2005). TRPM3 channel stimulation results in the activation of intracellular signalling cascades involving a rise in intracellular Ca^2+^ concentration ([Ca^2+^]_i_), activation of the protein kinases Raf, Extracellular signal- Regulated Kinases (ERK) and C-Jun N-terminal Kinases (JNK), and the activation of the stimulus-responsive transcription factors Activator Protein 1 (AP-1), C-AMP Response Element-binding protein (CREB), Early growth response protein 1 (Egr-1), and Elk-1 (Thiel et al., 2013). Therefore, understanding the mechanisms of the conversion of Ca^2+^ signalling into biological responses provides an exciting challenge in clinically relevant pathophysiology processes.

Activation of TRPM3 channels is often assessed by measuring either [Ca^2+^]_i_ with appropriate indicators and/or cationic membrane currents with a whole-cell patch-clamp technique (Lesch et al., 2014). Thus, the influx of Ca^2+^ into the cells and the subsequent rise in the [Ca^2+^]_i_ is used to demonstrate the activation of TRPM3 channels. TRPM3 is a polymodally activated channel that can be activated by both physical and chemical stimuli (Taberner et al., 1848). Several metabolites and synthetic compounds have been proposed to function as ligands for TRPM3 channels, including an endogenous neurosteroid pregnenolone sulfate (PregS) and a L-type voltage-gated Ca^2+^ channel inhibitor nifedipine (Wagner et al., 2008; Naylor et al., 2010; Islam, 2011). Experiments using stimulus-responsive transcription factors as a measure for activated TRPM3 channels showed that PregS is a powerful activator of TRPM3 channels (Lesch et al., 2014). In addition, studies performed with sensory neurons derived from TRPM3-deficient mice confirmed that TRPM3 is the major receptor for PregS (Vriens et al., 2011). On the other hand, the dihydropyridine nifedipine is an L-type Ca^2+^ channel blocker clinically used for the treatment of conditions such as cardiac arrhythmias, angina, hypertension, and preterm labor (Conde-Agudelo et al., 2011; Hirasawa & Pittman, 2003). *Wagner* et al.*,* reported that nifedipine paradoxically activates TRPM3 channels and triggers a rise of [Ca^2+^]_i_ in recombinant experiments and in pancreatic islets cells with a potency similar to that of PregS (Wagner et al., 2008; Majeed et al., 2011). However, PregS and nifedipine have entirely different chemical structures, and act on separate binding sites to quickly and reversibly activate TRPM3 channels (Wagner et al., 2008; Drews et al., 2014). Indeed, Drews et al. reported that co-application of PregS and nifedipine caused a larger activation of TRPM3 than applying these compounds alone (Drews et al., 2014), suggesting a ‘cocktail’ approach may have a role in therapy. PregS activates TRPM3 channels via binding to the extracellular side of the membrane (Wagner et al., 2008) on a stereo-specific binding site, the “steroid modulatory domain” (Thiel et al., 2017). However, this specific binding site has not been identified. Finally, Straub et al. reported that deoxybenzoin ononetin, a natural compound, is a selective and potent blocker of PregS- and nifedipine- induced TRPM3 currents in TRPM3-expressing dorsal root ganglia neurons and TRPM3 transfected HEK293 cells (Straub et al., 2013).

Previous studies suggested the importance of TRPM3 in the pathophysiology of CFS/ME. Five single nucleotide polymorphisms (SNPs) (rs6560200, rs1106948, rs12350232, rs11142822, rs1891301) have been identified in *TRPM3* genes in CFS/ME patients (Marshall-Gradisnik et al., 2016). Subsequently, TRPM3 expression was characterized on NK cells and B lymphocytes isolated from CFS/ME patients, in which it was found to have a significantly reduced cell surface expression of TRPM3 compared with healthy controls (HC) (Nguyen et al., 2016). Moreover, impaired Ca^2+^ mobilisation and reduced NK cell cytotoxicity in CFS/ME patients appeared to be associated with impaired TRPM3 channel activity (Nguyen et al., 2017). Finally, a recent electrophysiology investigation used whole-cell patch clamp techniques to report a loss of TRPM3 ion channel function in NK cells isolated from CFS/ME patients compared with HC after modulation with PregS and Ononetin (Cabanas et al., 2018). The ionic current evoked by PregS was significantly reduced in CFS/ME patients compared with HC. In addition, the PregS-induced ionic currents through TRPM3 were resistant to ononetin in CFS/ME patients when compared with HC, suggesting that TRPM3 ion channels are insensitive to ononetin or less expressed in CFS/ME patients (Cabanas et al., 2018). Collectively, these results suggests that TRPM3 activity is impaired in CFS/ME patients and may contribute to the pathophysiology of CFS/ME.

With the use of PregS and nifedipine as reversible activators of TRPM3 channels as well as ononetin as a potent blocker of PregS- and nifedipine- evoked Ca^2+^-influx and ionic currents, we aim to validate the previous research findings describing an impaired TRPM3 activity in a new cohort of CFS/ME patients using whole cell patch-clamp techniques. Moreover, no formal research has commented on using PregS or nifedipine to treat CFS/ME patients, however, there is evidence that physicians prescribe Ca^2+^ channel blockers to improve myalgia, orthostatic intolerance and cognitive symptoms (Carruthers et al., 2003; Chaudhuri et al., 2000). In conclusion, characterising the tissue-specific functions of TRPM3 channels in NK cells isolated from CFS/ME patients using pharmacological tools and whole-cell patch-clamp technique as the gold standard for ion channel research, will help to establish TRPM3 channels as a prognostic marker and/ or a potential therapeutic target for CFS/ME.

## Methods

### Participant recruitment

Six CFS/ME patients and six age- and sex-matched HC were recruited using the National Centre for Neuroimmunology and Emerging Diseases (NCNED) research database between October and December 2018. Participants were screened using a comprehensive questionnaire corresponding with the CDC, CCC and ICC case definitions. All six CFS/ME patients were defined by the CCC. HC reported no incidence of fatigue and were in good health without evidence of illness. Participants were excluded from this study if they were pregnant or breastfeeding, or reported a previous history of smoking, alcohol abuse or chronic illness (for example, autoimmune diseases, cardiac diseases and primary psychological disorders). No participants reported use of pharmacological agents that directly or indirectly influence TRPM3 or Ca^2+^ signalling. This investigation was approved by the Griffith University Human Research Ethics Committee (HREC/15/QGC/63).

### Peripheral blood mononuclear cell isolation and natural killer cell isolation

A total of 85 ml of whole blood was collected in ethylendiaminetetraacetic acid (EDTA) tubes between 8:30 am and 10:30 am on the Gold Coast, Queensland, Australia. Routine full blood analysis was performed within four hours of collection for red blood cell count, white blood cell count and granulocyte cell count.

Peripheral blood mononuclear cells (PBMCs) were isolated from 80 ml of whole blood by centrifugation over a density gradient medium (Ficoll-Paque Premium; GE Healthcare, Uppsala, Sweden) as previously described (Brenu et al., 2011b; Munoz & Leff, 2006). PBMCs were stained with trypan blue (Invitrogen, Carlsband, CA, USA) to determine cell count and cell viability. PBMCs were adjusted to a final concentration of 5 × 10^7^ cells/ml for NK cell isolation.

NK cells were isolated by immunomagnetic selection using an EasySep Negative Human NK Cell Isolation Kit (Stem Cell Technologies, Vancouver, BC, Canada). NK Cell purification was determined using flow cytometry. NK cells were incubated for 20 min at room temperature in the presence of CD56 FITC (0.25 μg/5 μl) and CD3 PE Cy7 (0.25 μg/20 μl) monoclonal antibodies (BD Bioscience, San Jose, CA, USA) as previously described (Nguyen et al., 2017). 7-amino-actinomycin (7-AAD) (2.5 μl/test) (BD Bioscience, San Jose, CA, USA) was used to determine cell viability. Cells were washed and resuspended in 200 μl of stain buffer (BD Bioscience, New Jersey, USA) and acquired at 10,000 events using the LSRFortessa X-20 (BD Biosciences, San Diego, CA, USA). Using forward and side scatter, the lymphocyte population was gated while acquiring the sample. The NK cell population was then identified as CD3^−^CD56^+^ cells.

### Whole cell electrophysiology recording

Borosilicate glass capillaries with an outside diameter of 1.5 mm and inside diameter of 0.86 mm (Harvard Apparatus, Holliston, MA, USA) were used as patch pipettes. Pipette resistance when filled with pipette solution was 8–12 MΩ. The pipettes were mounted on a CV203BU head-stage (Molecular Devices, Sunnyvale, CA, USA) connected to a 3-way coarse manipulator and a micro-manipulator (Narishige, Tokyo, Japan). Electrical signals were amplified and recorded using an Axopatch 200B amplifier and PClamp 10.7 software (Molecular Devices, Sunnyvale, CA, USA). Data were filtered at 5 kHz and sampled digitally at 10 kHz via a Digidata 1440A analogue to digital converter (Molecular Devices, Sunnyvale, CA, USA). The voltage-ramp protocol was a step from a holding potential of + 10 mV to − 90 mV, followed by a 0.1 s ramp to + 110 mV, before returning to + 10 mV (repeated every 10 s). The liquid junction potential between the pipette and bath solutions (− 10 mV) was corrected. A leak current component was not subtracted from the recorded currents. Electrode was filled with the intracellular pipette solution containing 30 mM CsCl, 2 mM MgCl_2_, 110 mM L-Aspartic acid, 1 mM EGTA, 10 mM HEPES, 4 mM ATP, 0.1 mM GTP, adjusted pH to 7.2 with CsOH and osmolality of 290 mOsm/L with D-mannitol. The pipette solution was filtered using a 0.22 μm membrane filter (Sigma-Aldrich, St. Louise, MO, USA), divided into aliquots and stored at − 20 °C. Bath solution contained: 130 mM NaCl, 10 mM CsCl, 1 mM MgCl_2_, 1.5 mM CaCl_2_2H_2_O, 10 mM HEPES, adjusted pH to 7.4 with NaOH and osmolality 300 mOsm/L with D-glucose. All reagents were purchased from Sigma-Aldrich, except for ATP and GTP that were purchased from Sapphire Bioscience. TRPM3 currents were stimulated by adding 100 μM PregS (Tocris Bioscience, Bristol, UK) or 100 μM nifedipine (Sapphire Bioscience, NSW, Australia) to the bath solution, whereas PregS- and nifedipine-induced TRPM3 currents were blocked by adding 10 μM ononetin (Tocris Bioscience, Bristol, UK). All measurements were performed at room temperature. The authors removed the possibility of chloride current involvement in TRPM3 assessment by using L-Aspartic acid in the intracellular pipette solution. Cells which have unstable currents were also excluded from the analysis.

### Statistical analysis

**Fig. 1:**
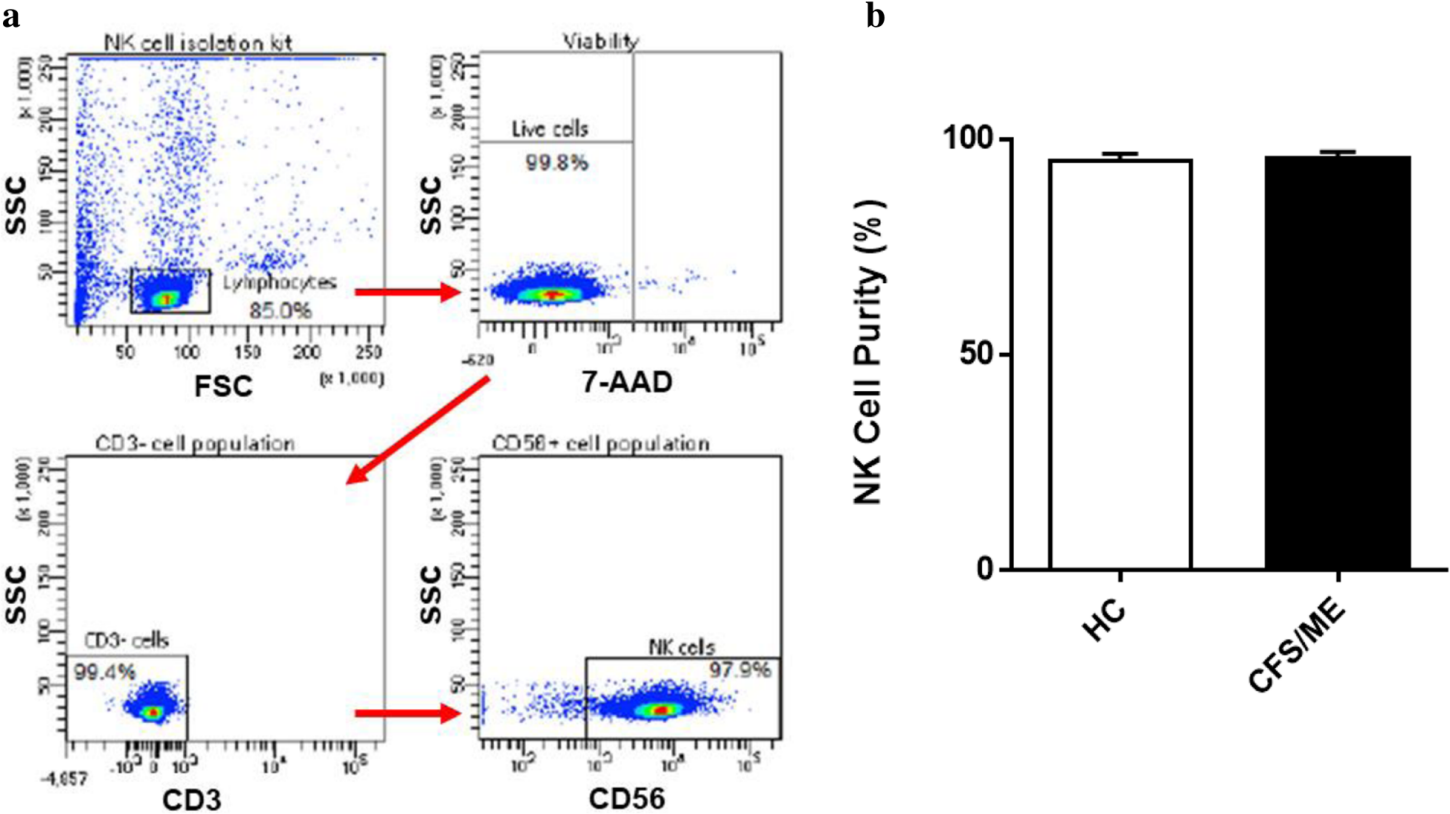
Natural Killer cell purity. **a** Gating strategy used to identify NK cells. Representative flow cytometry plots from the PBMCs of one of the study participants. The lymphocytes were live gated during acquisition using the side and forward scatter dot plot display and then single and dead cells were excluded. Furthermore, by using the negative and positive gating strategies, CD3^−^ as well as CD56^+^ lymphocyte populations were identified. **b** Bar graphs representing isolated NK cell purity for HC and CFS/ME patients. Data presented as mean ± SEM. HC = 95.3% ± 1.322 and CFS/ME = 95.98% ± 1.093. *Abbreviations: 7-AAD, 7-amino-actinomycin; CFS/ME, chronic fatigue syndrome/myalgic encephalomyelitis; FSC, forward scatter; HC, healthy controls; NK cell, natural killer cell; SSC, side scatter*

Lymphocyte populations were identified using forward and side scatter dot plots. Exclusions were CD3^+^ cells and only CD3^−^ lymphocytes were further used to characterise NK cell subset populations using CD56 and CD16. NK cell subsets were characterised using the surface expression of CD56^Bright^CD16^Dim/−^ NK cells and CD56^Dim^CD16^Bright/+^ NK cells. Cytometry data was exported from FacsDiva v8.1 and analysed using SPSS v24 (IBM Corp, Version 24, Armonk, NY, USA) and GraphPad Prism v7 (GraphPad Software Inc., Version 7, La Jolla, CA, USA). Electrophysiological data were analysed using pCLAMP 10.7 software (Molecular Devices, Sunnyvale, CA, USA). Origin 2018 (OriginLab Corporation, Northampton, MA, USA) and GraphPad Prism v7 (GraphPad Software Inc., Version 7, La Jolla, CA, USA) were used for statistical analysis and data presentation. Shapiro-Wilk normality tests were conducted to determine the distribution of data, in addition to skewness and kurtosis tests to determine data normality. Statistical comparison was performed using the independent Mann-Whitney U test (Table 1, Fig.1, and Fig.), and Fishers exact test (Fig.), to determine any significant differences. Significance was set at *p* < 0.05 and the data are presented as mean ± SEM unless otherwise stated.

**Table 1:** Blood parameters and patient demographic. SF-36 scores were analysed using participant questionnaire responses. Results from routine full blood analysis in CFS/ME patients and HC

	CFS/ME	HC	*P* Value
Age (years)	42.5 ± 3.5	43.5 ± 3.9	0.699
Gender n(%)			
Male	1	1	1.000
Female	5	5	
BMI (kg/m^2^)	24.04 ± 0.74	23.8 ± 1.46	0.589
WHODAS	47.4 ± 6.47	4.34 ± 3.68	0.002
SF-36			
General Health (%)	28.47 ± 5.63	75.69 ± 3.3	0.002
Physical Functioning (%)	35.83 ± 8.89	95.83 ± 1.54	0.002
Role Physical (%)	5.27 ± 4.09	97.92 ± 2.08	0.002
Role Emotional (%)	70.48 ± 15.32	100 ± 0	0.180
Social Functioning (%)	35.42 ± 13.85	98 ± 2	0.030
Body Pain (%)	37 ± 14.12	94.58 ± 2.45	0.004
Pathology			
White Cell Count (× 10^9^/L)	6.18 ± 0.38	6.15 ± 0.51	1.000
Lymphocytes (× 10^9^/L)	2.11 ± 0.22	1.85 ± 0.10	0.132
Neutrophils (× 10^9^/L)	3.24 ± 0.28	3.62 ± 0.10	0.589
Monocytes (× 10^9^/L)	0.52 ± 0.03	0.48 ± 0.08	0.699
Eosinophils (× 10^9^/L)	0.26 ± 0.10	0.15 ± 0.03	0.394
Basophils (× 10^9^/L)	0.05 ± 0.01	0.05 ± 0.01	0.937
Platelet (× 10^9^/L)	237 ± 8.99	269.33 ± 16.64	0.180
Red Cell Count (× 10^12^/L)	4.49 ± 0.17	4.80 ± 0.31	0.485
Haematocrit	0.40 ± 0.01	0.42 ± 0.02	0.394
Haemoglobin (g/L)	137.17 ± 5.88	140.83 ± 5.17	0.485

Data presented as mean ± SEM. *Abbreviations: CFS/ME, chronic fatigue syndrome/myalgic encephalomyelitis; HC, healthy controls; BMI, body mass index; WHODAS: World Health Organization Disability Assessment Schedule*

## Results

### Participant characteristics and blood parameters

Twelve age- and sex-matched participants were included for this investigation. Demographic and clinical data for patients are summarised in Table 1. There was no significant difference in age or gender between patients and HC. The 36-Item Short Form Survey (SF-36) and World Health Organization Disability Assessment Schedule (WHODAS) were used to determine participant health-related-quality of life (Andrews et al., 2009; SF-36 interim norms for Australian data, Summary, 2019). As expected, there was a significant difference in SF-36 and WHODAS scores between CFS/ME patients and HC. Moreover, full blood count parameters were measured for each healthy participant. All participant results were within the specified reference ranges for each parameter. There were no significant differences between healthy participants and CFS/ME patients for these reporting parameters as provided by the Gold Coast University Hospital Pathology Unit, NATA accredited laboratory.

### Natural killer cell purity

The NK cell population was identified as CD3^−^CD56^+^ cells by flow cytometry (Fig. 1a). NK cell purity was 95.3% ± 1.322 for HC and 95.98% ± 1.093 for CFS/ME patients (Fig. 1b). There was no significant difference in NK cell purity in CFS/ME patients compared with HC.

### Effects of successive applications of PregS and nifedipine on TRPM3 activity

**Fig. 2:**
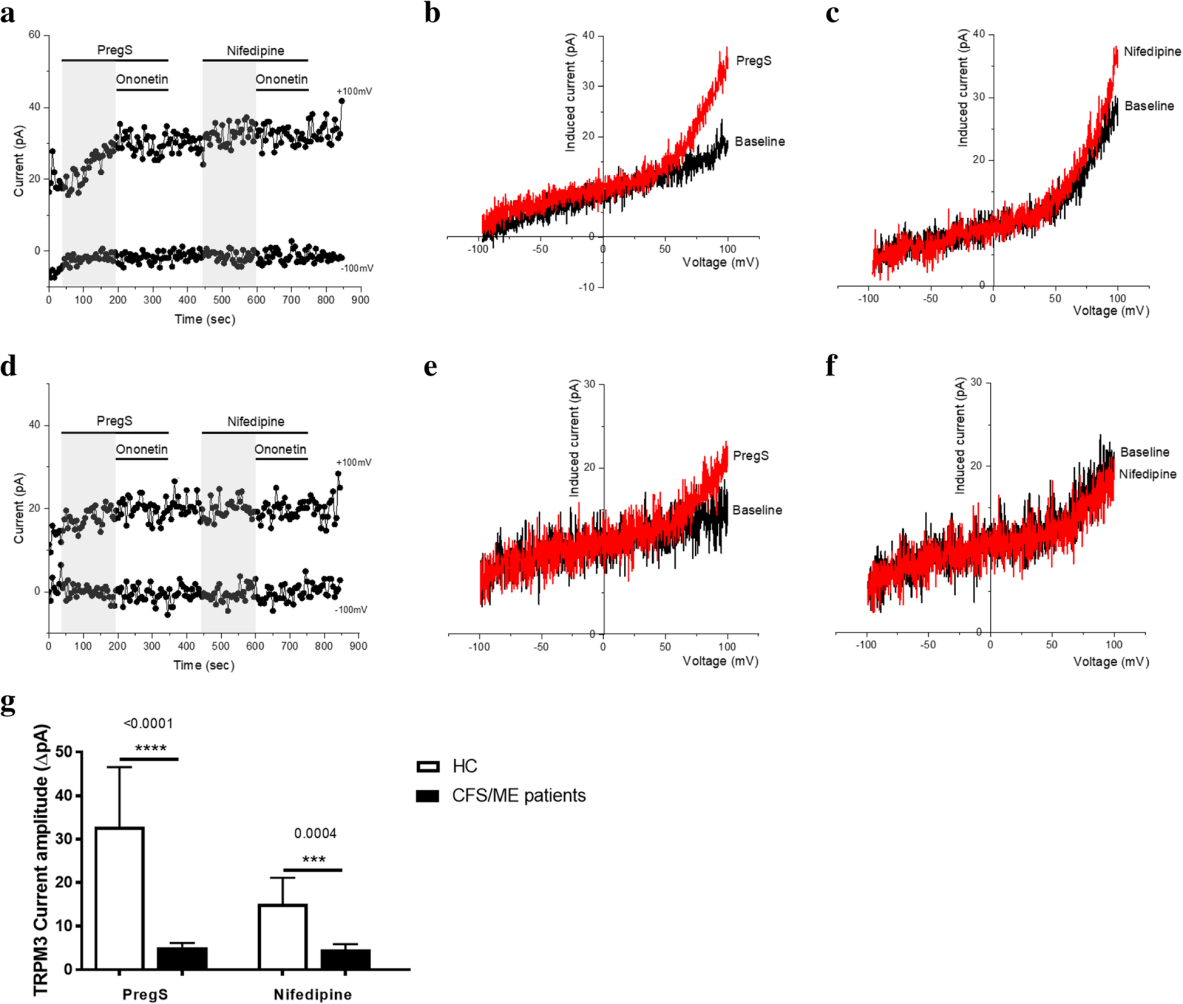
TRPM3 activity after successive applications of PregS and nifedipine. Data were obtained under whole-cell patch clamp conditions. **a.** A representative time-series of current amplitude at + 100 mV and − 100 mV showing the effect of 100 μΜ PregS and 100 μM nifedipine on ionic currents in isolated NK cells from HC. **b.** *I*–*V* before and after PregS stimulation in a cell corresponding with (**a**.)**. c.** *I*–*V* before and after nifedipine stimulation in a cell corresponding with (**a**.)**. d.** A representative time-series of current amplitude at + 100 mV and − 100 mV showing the effect of 100 μΜ PregS and 100 μM nifedipine on ionic currents in isolated NK cells from CFS/ME patients. **e.** *I*–*V* before and after PregS stimulation in a cell as shown in (**d**.)**. f.** *I*–*V* before and after nifedipine stimulation in a cell as shown in (**d**.)**. g.** Bar graphs representing TRPM3 current amplitude at + 100 mV after stimulation with 100 μΜ PregS and 100 μM nifedipine in CFS/ME patients (*N = 6*; *n* = 24) compared with HC (N = 6; *n* = 18). Data are represented as mean ± SEM. *Abbreviations: CFS/ME, chronic fatigue syndrome/myalgic encephalomyelitis; HC, healthy controls; NK, natural killer; PregS, Pregnenolone sulfate; TRPM3, Transient Receptor Potential Melastatin 3*

Patch-clamp remains the most direct technique to study the properties of ion channels. To confirm the impaired TRPM3 activity in CFS/ME patients, we performed whole-cell patch clamp measurements in NK cells isolated from HC and CFS/ME patients. Endogenous TRPM3 channels were stimulated by a first application of 100 μM PregS and a successive application of 100 μM nifedipine to the extracellular side via the bathing solution (Fig. 2a and d). We measured a small outwardly rectifying current under voltage-clamp conditions with a typical shape of the TRPM3 current–voltage relationship (*I*–*V*) in NK cells isolated from HC after addition of PregS (Fig. 2b). As previously shown (Cabanas et al., 2018), the outward current amplitudes decreased dramatically and significantly after PregS stimulation in NK cells from CFS/ME patients (Fig. 2e and g) (*p* < 0.0001). To confirm that the currents evoked by PregS are sensitive to nifedipine, another reversible TRPM3-agonist, we then stimulated the NK cells with nifedipine. Similar increased in TRPM3-like currents was observed after the application of nifedipine. As shown in Fig. 2c, although the ionic current evoked by nifedipine was relatively small in NK cell isolated from HC, the current–voltage relationship (*I-V*) of nifedipine-induced currents had a clear TRPM3-like outward rectification. In contrast, the amplitude of ionic current after nifedipine stimulation was significantly smaller in NK cells from CFS/ME patients (Fig. 2f and g) (*p* = 0.0004) showing that the profile described after PregS stimulation represents TRPM3 activity. These results confirm that TRPM3 channel activity is impaired after nifedipine as well as PregS stimulation in CFS/ME patients.

### Modulation of PregS- and nifedipine- evoked currents with Ononetin

**Fig. 3:**
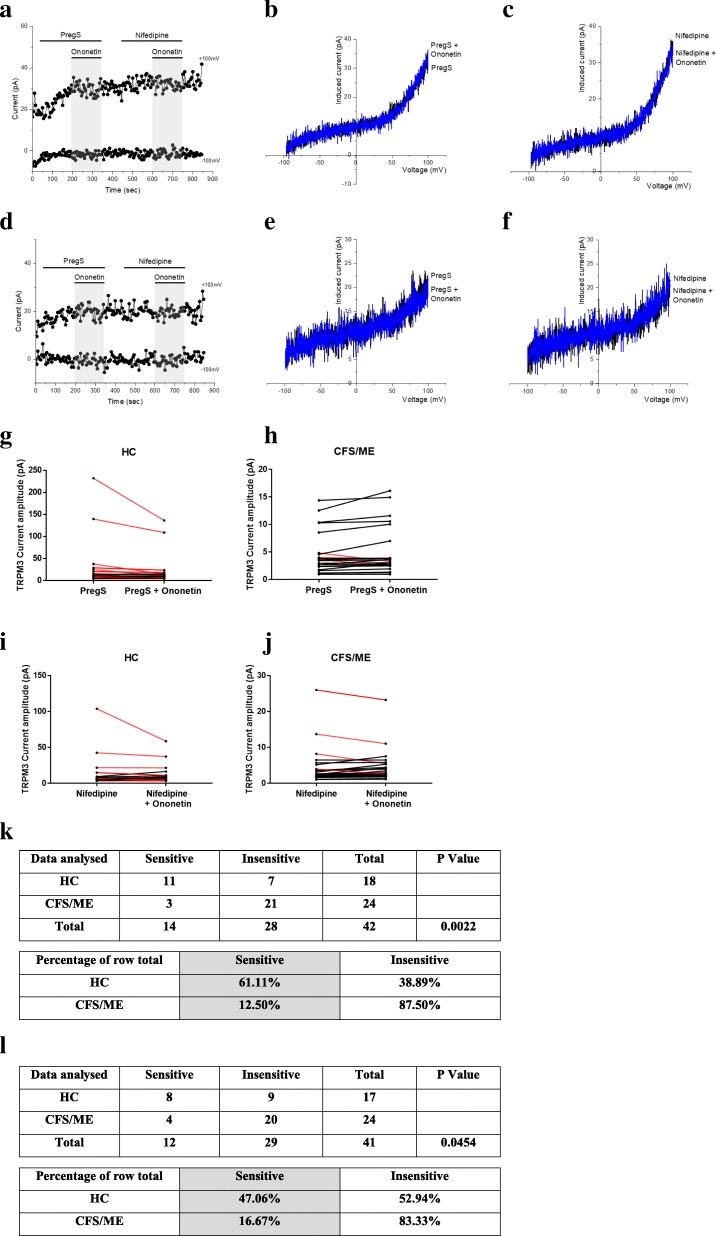
Modulation of PregS- and nifedipine- evoked currents with Ononetin**.** Data were obtained under whole-cell patch clamp conditions. **a.** A representative time-series of current amplitude at + 100 mV and − 100 mV showing the effect of 10 μΜ ononetin on ionic currents in the presence of PregS or nifedipine in isolated NK cells from HC. **b.** *I*–*V* before and after application of ononetin in the presence of PregS in a cell as shown in (**a**.)**. c.** *I*–*V* before and after application of ononetin in the presence of nifedipine in a cell as shown in (**a**.)**. d.** A representative time-series of current amplitude at + 100 mV and − 100 mV showing the effect of 10 μΜ ononetin on ionic currents in the presence of PregS or nifedipine in isolated NK cells CFS/ME patients. **e.** *I*–*V* before and after application of ononetin in the presence of **PregS** in a cell as shown in (**d**.). **f.** *I*–*V* before and after application of ononetin in the presence of nifedipine in a cell as shown in (**d**.)**. g.h.** Scatter plots representing change of each current amplitude before and after ononetin application in presence of PregS in all NK cells from HC and CFS/ME patients. Each cell represented as red lines had reduction in currents by ononetin. **i.j.** Scatter plots representing change of each current amplitude before and after ononetin application in presence of nifedipine in all NK cells from HC and CFS/ME patients. Each cell represented as red lines had reduction in currents by ononetin. **k.** Table summarizing data for sensitive and insensitive cells to 10 μΜ ononetin in presence of PregS in HC (*N* = 6; *n* = 18) compared to CFS/ME patients (N = 6; *n* = 24). l. Table summarizing data for sensitive and insensitive cells to 10 μΜ ononetin in presence of nifedipine in HC (N = 6; *n* = 17) compared to CFS/ME patients (N = 6; *n* = 24). Data are analysed with Fisher’s exact test. *Abbreviations: CFS/ME, chronic fatigue syndrome/myalgic encephalomyelitis; HC, healthy controls****;*** *NK, natural killer; PregS, Pregnenolone sulfate; TRPM3, Transient Receptor Potential Melastatin 3*

Ononetin effectively inhibits PregS- and nifedipine- evoked Ca^2+^-influx and ionic currents through TRPM3 channels (Straub et al., 2013). Therefore, to confirm that TRPM3 activity is involved in ionic currents evoked by PregS and nifedipine in NK cells, we next used 10 μM ononetin to modulate the channels (Fig. 3). As previously shown (Cabanas et al., 2018), the ionic currents evoked by PregS were effectively inhibited by simultaneous application of ononetin in isolated NK cells from HC (Fig. 3a and g). As expected, ionic currents in the presence of PregS were mostly resistant to ononetin in isolated NK cells from CFS/ME patients (Fig. 3d and h), in comparison with HC (*p* = 0.0022). Interestingly, similar results were obtained with the nifedipine. Indeed, the inhibitory effect of ononetin on nifedpine- evoked ionic currents through TRPM3 channels was not significantly different from that observed with PregS-evoked TRPM3 activity in HC (Fig. 3g and i).. Finally, we also observed a resistance to ononetin in isolated NK cells from CFS/ME patients (Fig. 3d, f, i, j and l) (*p* = 0.0454). Collectively, these results confirm the significant loss of the TRPM3 channel activity in CFS/ME patients.

### TRPM3 activity after stimulation with nifedipine alone

**Fig. 4:**
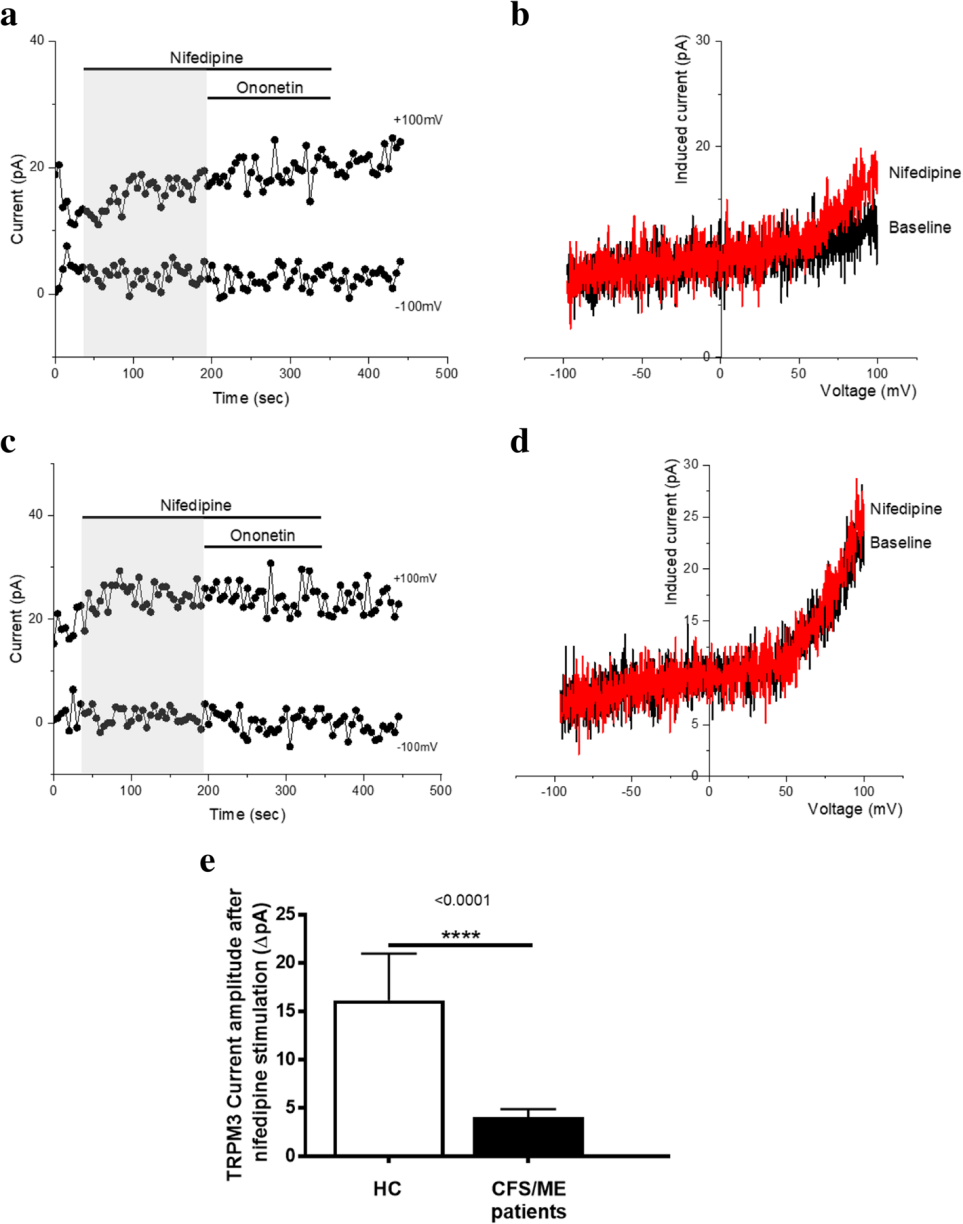
TRPM3 activity after stimulation with nifedipine alone. Data were obtained under whole-cell patch clamp conditions. **a.** A representative time-series of current amplitude at + 100 mV and − 100 mV showing the effect of 100 μΜ nifedipine on ionic currents in isolated NK cells from HC. **b.** *I*–*V* before and after nifedipine stimulation in a cell corresponding with (**a**.)**. c.** A representative time-series of current amplitude at + 100 mV and − 100 mV showing the effect of 100 μΜ nifedipine on ionic currents in isolated NK cells from CFS/ME patients. **d.** *I*–*V* before and after nifedipine stimulation in a cell as shown in (**c**.)**. e** Bar graphs representing TRPM3 current amplitude at + 100 mV after stimulation with 100 μΜ nifedipine in CFS/ME patients (*N* = 6; *n* = 25) compared with HC (N = 6; *n* = 23). Data are represented as mean ± SEM. *Abbreviations: CFS/ME, chronic fatigue syndrome/myalgic encephalomyelitis; HC, healthy controls****;*** *NK, natural killer; PregS, Pregnenolone sulfate; TRPM3, Transient Receptor Potential Melastatin 3*

Nifedipine is chemically distinct from PregS and activates TRPM3 on a separate binding site (Wagner et al., 2008; Thiel et al., 2017). Therefore, to further confirm our previous finding that TRPM3 channel activity is impaired in CFS/ME patients, we directly stimulated TRPM3 in NK cells using only the non-steroidal L-type Ca^2+^ channel blocker agent nifedipine. We tested whether the amplitudes of the nifedipine-induced currents were decreased in NK cells isolated from CFS/ME patients. Nifedipine (100 μM) evoked small ionic currents (Fig. 4.a), which is expected in NK cells isolated from HC. Moreover, as a TRPM3 agonist, the stimulation of nifedipine mimicked the PregS-induced increase, but with an expected reduction in potency and efficacy as PregS is currently the most potent TRPM3 agonist described in the literature (Held et al., 2015). The *I-V* of the nifedipine-evoked currents was outwardly rectified (Fig. 4. b), which is standard for TRPM3. Finally, as previously shown with PregS stimulation, the outward current amplitudes decreased dramatically and significantly after nifedipine stimulation in NK cells from CFS/ME patients (Fig. 4c, d and e) (*p* < 0.0001). The data suggest that TRPM3 channel activity after nifedipine stimulation is also impaired in CFS/ME patients, confirming that low TRPM3 activity is involved in ionic currents in NK cells.

### TRPM3 activity after co-modulation with nifedipine and ononetin

**Fig. 5:**
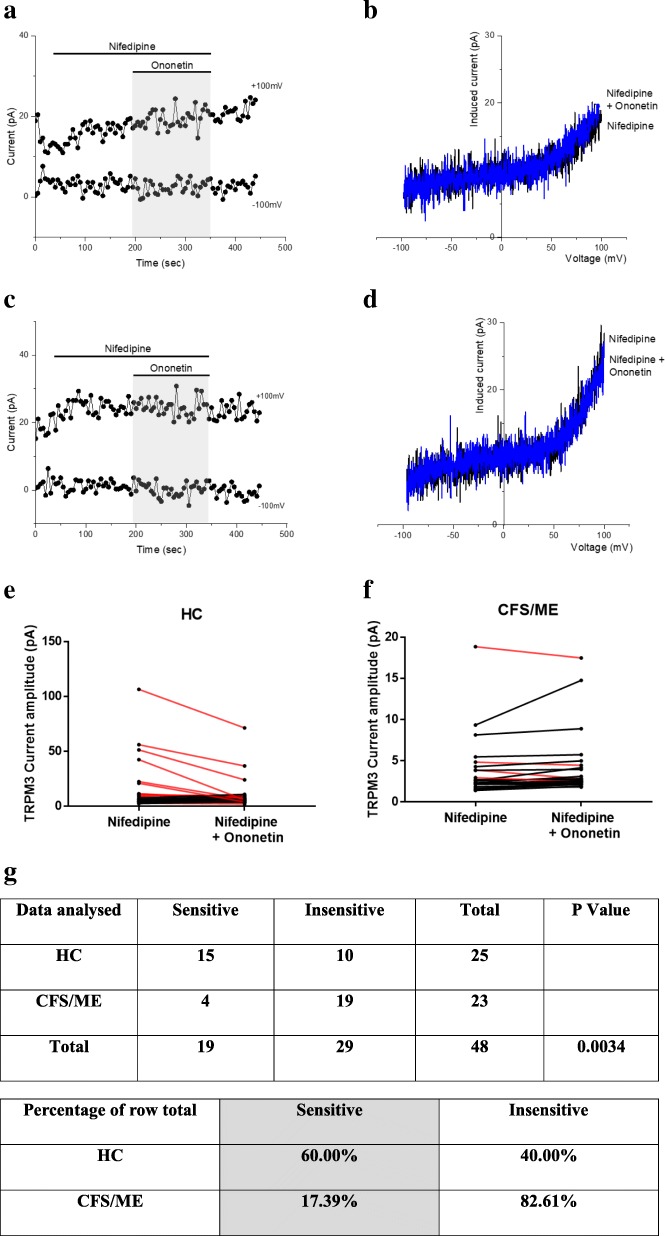
TRPM3 activity after co-modulation with nifedipine and ononetin. Data were obtained under whole-cell patch clamp conditions. **a.** A representative time-series of current amplitude at + 100 mV and − 100 mV showing the effect of 10 μΜ ononetin on ionic currents in the presence of nifedipine in isolated NK cells from HC. **b.** *I*–*V* before and after application of ononetin in a cell as shown in (**a**.)**. c.** A representative time-series of current amplitude at + 100 mV and − 100 mV showing the effect of 10 μΜ ononetin on ionic currents in the presence of nifedipine in isolated NK cells CFS/ME patients. **d.** *I*–*V* before and after application of ononetin in a cell as shown in (**c**.)**. e. f.** Scatter plots representing change of each current amplitude before and after ononetin application in all NK cells from HC and CFS/ME patients. Each cell represented as red lines had reduction in currents by ononetin. **g.** Table summarizing data for sensitive and insensitive cells to 10 μΜ ononetin in HC (N = 6; *n* = 25) compared to CFS/ME patients (N = 6; *n* = 23). Data are analysed with Fisher’s exact test. *Abbreviations: CFS/ME, chronic fatigue syndrome/myalgic encephalomyelitis; HC, healthy controls****;*** *NK, natural killer; PregS, Pregnenolone sulfate; TRPM3, Transient Receptor Potential Melastatin 3*

As shown in Fig. 5. a and e, the ionic currents evoked by nifedipine were effectively inhibited by simultaneous application of 10 μM ononetin in isolated NK cells from HC. In contrast, ionic currents in the presence of nifedipine were mostly resistant to ononetin in isolated NK cells from CFS/ME patients (Fig. 5. c and f), in comparison with HC, showing significant loss of the TRPM3 channel activity after nifedipine stimulation in CFS/ME patients.

## Discussion

Whole-cell patch-clamp technique was used as a method to measure endogenous TRPM3 activity in isolated NK cells from HC and CFS/ME patients, enabling the ion channel current recordings under voltage-clamp conditions and observation of the shape of the TRPM3 current–voltage relationship (*I*–*V*). This present study has again confirmed the significant loss of the TRPM3 channel activity after PregS stimulation and ononetin modulation in CFS/ME patients. Moreover, similar results have been reported when testing nifedipine after PregS stimulation as well as stimulation with nifedipine alone. Indeed, the use of this agonist has allowed us to further investigate TRPM3 activity in isolated NK cells from HC and CFS/ME patients. More precisely, we demonstrated that the amplitude of ionic current after PregS stimulation and either successive or independent nifedipine stimulation was significantly smaller in NK cells from CFS/ME patients than that from HC, confirming an impaired TRPM3 channel activity in CFS/ME patients. Moreover, we found both PregS- and nifedipine- evoked ionic currents through TRPM3 ion channels were significantly modulated by ononetin in isolated NK cells from HC in comparison with CFS/ME patients. Indeed, ionic currents in the presence of PregS or nifedipine were mostly resistant to ononetin in isolated NK cells from CFS/ME patients suggesting that constitutive TRPM3 cationic currents may be not expressed in CFS/ME patients. Alternatively, the severity and nature of Ca^2+^ signalling perturbation may depend upon the isoforms and extent of TRPM3 ion channels affected that may be non-sensitive to the different pharmacological tools used in this study.

The neurosteroid PregS induces rapid and reversible activation of TRPM3, both in overexpression systems and in cells endogenously expressing TRPM3, and PregS is currently the most potent TRPM3 agonist described in the literature (Vriens et al., 2011; Wagner et al., 2008; Held et al., 2015; Straub et al., 2013; Klose et al., 2011). PregS is a substance extensively produced by the human body, although the conditions under which elevated PregS levels may gate TRPM3 are not known (Havlíková et al., 2002). Interestingly, TRPM3 is more sensitive to PregS when increasing the temperature from room temperature to 37 °C, and it is has been proposed that the PregS concentrations encountered physiologically in the human body may be sufficient to activate TRPM3 channels, especially at the body core temperature (Vriens et al., 2011; Wagner et al., 2008; Harteneck, 2013). In addition, PregS may also be used in a medical context as an adrenal cortex hormone, glucocorticoid, and anti-inflammatory for indications such as the treatment of rheumatoid arthritis (Ciurtin et al., 2010). PregS may also be prescribed to improve symptoms of schizophrenia as well as to help fight fatigue, increase energy, enhance memory, and improve mood (Wong et al., 2015). Indeed, PregS has multiple important effects on brain functions, such as cognitive enhancing, promnesic, antistress, and antidepressant effects, by regulating the release of many important neurotransmitters including glutamate, *gamma-aminobutyric acid (*GABA), acetylcholine, norepinephrine, and dopamine (Wong et al., 2015). On the other hand, the 1,4-dihydropyridine nifedipine is another reversible TRPM3 agonist (Held et al., 2015). Nifedipine is clinically used to treat high blood pressure and to control angina (chest pain) (Terry, 1982; Snider et al., 2008). *Nifedipine also* helps to prevent future heart disease, heart attacks and strokes. No formal research has commented on using PregS or nifedipine to treat CFS/ME patients, however, there is evidence that physicians prescribe Ca^2+^ channel blockers to improve myalgia, orthostatic intolerance and cognitive symptoms (Carruthers et al., 2003; Chaudhuri et al., 2000b). As PregS and nifedipine act on TRPM3 via separate binding sites (Drews et al., 2014), since co-application of PregS and nifedipine caused a larger activation of TRPM3 than applying these compounds alone, a drugs combination approach may be considered for CFS/ME therapy. However, further studies are required to establish whether PregS or nifedipine, as well as a drugs combination, may be a possible therapeutic approach for CFS/ME patients in order to restore the TRPM3 ion channel function.

TRPM3 stimulation by heat or chemical compounds, like PregS and nifedipine, opens the central pore and induces outwardly rectifying currents in TRPM3-expressing cells (Oberwinkler et al., 2005; Cabanas et al., 2018; Held et al., 2015). The central pore is highly permeable for Ca^2+^ and magnesium (Mg^2+^). Ca^2+^ is an ubiquitous second messenger with wide-ranging physiological roles including cell differentiation and division, apoptosis, and transcriptional events (Berridge, 2012). In NK cells, Ca^2+^ regulates cytotoxic activity by driving the intracellular microtubule reorganisation, polarisation of cytoplasmic granules, production, recruitment and release of lytic proteins, creation of the immune synapse, formation of perforin pores and granzyme-induced cell apoptosis (Anasetti et al., 1987; Henkart, 1985; Voskoboinik et al., 2015; Orrenius et al., 2003; Voskoboinik et al., 2005). In non-excitable cells, such as immune cells, store-operated Ca^2+^ entry (SOCE) is a major Ca^2+^ entry pathway, which involves several steps for activation, including stimulation of G proteins or protein tyrosine kinases; activation of phospholipase C; hydrolyse of phosphatidylinositol 4,5-biphosphate (PIP_2_) and release of the second messenger inositol-1, 4, 5-trisphosphate (IP_3_); binding of IP_3_ to its receptor in the endoplasmic reticulum (ER) membrane; rapid and transient Ca^2+^ release from ER lumen; and finally activation of SOCE in the plasma membrane (Prakriya & Lewis, 2015). TRP ion channels are candidate cation entry channels in this important cellular mechanism. The sub-family TRPC is traditionally associated in this signalling pathway (Cheng et al., 2013; Ong et al., 2016; Salido et al., 2009), however, a recent research has also identified TRPM3 as a component for SOCE in white matter of the central nervous system (CNS) (Papanikolaou et al., 2017). Upon TRPM3 channel activation, changes in [Ca^2+^]_i_ occur, resulting in the activation of an intracellular signalling cascade including the protein kinases Raf, ERK and JUN and the stimuli-responsive transcription factors AP-1, CREB, Egr-1 and Elk-1. Activation of TRPM3 is critical to regulate various physiological processes that correspond to an array of cells expressing this channel (Thiel et al., 2013; Thiel et al., 2017). TRPM3 is located and linked to vascular smooth muscle contraction, modulation of glucose-induced insulin release from pancreatic beta-cells, detection of noxious heat in dorsal root ganglia and development of epithelial cells of the choroid plexus, as well as function of oligodendrocytes and neurons (Vriens et al., 2011; Hoffmann et al., 2010; Wagner et al., 2008; Oberwinkler et al., 2005). Therefore, dysregulation of TRPM3 family, affecting SOCE and more generally, Ca^2+^ signalling has significant implications for cell regulatory machinery and represents a novel and attractive therapeutic target of TRP pathology.

Numerous and significant anomalies including genetic, proteomic and functional anomalies in TRPM3 ion channels have been reported in CFS/ME patients suggesting perturbations of Ca^2+^ signalling in NK cells from CFS/ME patients. Indeed, a previous study reported indicative evidence of SNPs in *TRPM3* genes from isolated peripheral blood mononuclear cells, NK and B cells in patients with CFS/ME and have been proposed to correlate with illness presentation (Marshall-Gradisnik et al., 2016). The most consistent feature reported in CFS/ME is a reduction in NK cell cytotoxicity (Brenu et al., 2012; Curriu et al., 2013; Hardcastle et al., 2015; Huth et al., 2016; Klimas et al., 1990; Maher et al., 2005; Natelson et al., 2002; Nijs & Frémont, 2008; Sharpe et al., 1991; Siegel et al., 2006; Stanietsky & Mandelboim, 2010; Brenu et al., 2011b), which is a Ca^2+^ dependent process (Anasetti et al., 1987; Henkart, 1985). Impaired Ca^2+^ signalling in NK cells from CFS/ME patients has been demonstrated through changes to ERK1/2 and mitogen-activated protein kinase (MAPK) pathways (Chacko et al., 2016; Huth et al., 2016), as these components are activated in a Phosphatidylinositol-4,5-bisphosphate 3-kinase (P13K)-dependent manner that may also be associated with cytoplasmic Ca^2+^ ion levels through activation of TRPM3 (Lee et al., 2003). Indeed, TRPM3 is directly regulated by PIP_2_ to result in a rise of [Ca^2+^]_i_ (Tóth et al., 2015). Moreover, a significant reduction in both TRPM3 surface expression and intracellular Ca^2+^ mobilisation in NK cells has been found in CFS/ME patients compared with HC (Nguyen et al., 2016; Nguyen et al., 2017). The modification of Ca^2+^ concentration in the cytosol and intracellular stores may thereby alter the activation threshold of NK cells and their activity as ERK1/2 requires Ca^2+^ as the final activator to initiate NK cell lysis (Huth et al., 2016). Finally, we recently reported a TRPM3 ion channel dysfunction in NK cells from CFS/ME patients (Cabanas et al., 2018) and confirmed these results in the present study. Therefore, TRPM3-related Ca^2+^ dysfunction may then result in a reduction of [Ca^2+^]i, which may lower the function and cytotoxic capacity of the NK cells in CFS/ME patients. Changes in Ca^2+^ signalling may also impair cytokine production, including Interferon (IFN)-γ and Tumor Necrosis Factor (TNF), therefore interfering with systemic inflammation and anti-tumour responses (Romee et al., 2013). Collectively these significant findings highlight the potential role of TRPM3 ion channels in the aetiology and pathomechanism of CFS/ME, in addition to possible pharmacological targets and/ or prognostic markers. However, further investigations are required to elucidate the mechanisms involved in the impaired TRPM3 channel activity as well as the different TRPM3 isoform types that are expressed in NK cells.

Previous studies have already suggested TRPM channels as potential therapeutic targets. *Zierler* et al., noted that mutations in TRPM cation channels play a role in pro-inflammatory diseases and therefore are potential drug targets indicating the roles of TRP-Ca^2+^ pathways in pathophysiological immune responses (Zierler et al., 2017). *Schattling* et al.*,* demonstrated that TRPM4 contributes to axonal and neuronal injury under inflammatory conditions in experimental autoimmune encephalomyelitis in mice and in human multiple sclerosis tissue (Schattling et al., 2012). Indeed, the deficiency or pharmacological inhibition of TRPM4 using the antidiabetic drug glibenclamide, resulted in reduced axonal and neuronal degeneration. Importantly, TRP ion channels have a role in heat detection and pain mediation in the CNS and hence are targets for analgesic pharmaco-therapeutics (Vriens et al., 2011; Held et al., 2015). *Dembla* et al.*,* have reported that TRPM3 inhibition is an important consequence of peripheral μ-opioids receptors activation indicating that pharmacologically antagonizing TRPM3 may be a useful analgesic strategy (Dembla et al., 2017). Finally, dysregulation of thermoregulatory responses has been reported in CFS/ME patients (Wyller et al., 2007). Generalised pain is a characteristic of CFS/ME and occurs in the absence of other tissue damage, and this is suggestive of potential CNS impairments (Barnden et al., 2015; Barnden et al., 2016; Shan et al., 2016; Shan et al., 2017). Therefore, our present findings suggest TRPM3 ion channels may be involved in the pathomechansim of CFS/ME and hence have a possible role in nociception and thermoregulation.

## Conclusions

We have confirmed impaired TRPM3 activity in CFS/ME patients through electrophysiological investigations in NK cells after both successive and independent activation with PregS and nifedipine and inhibition with ononetin. This study not only helps to explain the impaired NK cellular functions observed in CFS/ME patients, but helps to provide an understanding of the aetiology and pathomechanism of this illness. Finally, the discovery of TRPM3 ion channel dysfunctions contributing to the pathomechanism of CFS/ME paves the way for investigating possible pharmacological treatments through functional investigations in the NK cell model which may lead to drug repurposing or drug development de novo.

## Abbreviations

[Ca^2+^]_i_: Intracellular Ca^2+^ concentration
AP-1: Activator Protein 1
BMI: Body Mass Index
Ca^2+^: Calcium
CCC: Canadian Consensus Criteria
CDC: Centers for Disease Control and Prevention
CFS/ME: Chronic Fatigue Syndrome/Myalgic Encephalomyelitis
CNS: Central nervous system
CREB: C-AMP Response Element-binding protein
EDTA: Ethylendiaminetetraacetic acid
Egr-1: Early growth response protein 1
ER: Endoplasmic reticulum
ERK: Extracellular signal-regulated kinases
GABA: *Gamma-aminobutyric acid*
HC: Healthy controls
ICC: International Consensus Criteria
IFN: Interferon
IP_3_: Inositol-1, 4, 5-trisphosphate
JNK: C-Jun N-termianl Kinases
MAPK: Mitogen-activated protein kinase
Mg^2+^: Magnesium
NCNED: National Centre for Neuroimmunology and Emerging Diseases
NK: Natural killer
PBMCs: Peripheral blood mononuclear cells
PI_3_K: Phosphatidylinositol-4, 5-bisphosphate 3-kinase
PIP_2_: Phosphatidylinositol 4,5-bisphosphate
PregS: Pregnenolone sulfate
SF-36: 36-Item Short Form Survey
SNPs: Single nucleotide polymorphisms
SOCE: Store-operated Ca^2+^ entry
TNF: Tumour necrosis factor
TRP: Transient Receptor Potential
TRPA: Transient Receptor Potential Ankyrin
TRPC: Transient Receptor Potential Canonical
TRPM: Transient Receptor Potential Melastatin
TRPML: Transient Receptor Potential Mucolipin
TRPP: Transient Receptor Potential Polycystin
TRPV: Transient Receptor Potential Vanilloid
WHODAS: World Health Organization Disability Assessment Schedule
